# The Effectiveness of Teaching Nursing Ethics via Scenarios and Group Discussion in Nurses' Adherence to Ethical Codes and Patients' Satisfaction with Nurses' Performance

**DOI:** 10.1155/2020/5749687

**Published:** 2020-06-01

**Authors:** Fatemeh Izadi, Mostafa Bijani, Zhila Fereidouni, Shahnaz Karimi, Banafsheh Tehranineshat, Azizallah Dehghan

**Affiliations:** ^1^Fasa University of Medical Sciences, Fasa, Iran; ^2^Department of Medical Surgical Nursing, Fasa University of Medical Sciences, Fasa, Iran; ^3^Department of Nursing, School of Nursing and Midwifery, Fasa University of Medical Science, Fasa, Iran; ^4^Department of Nursing, School of Nursing and Midwifery, Shiraz University of Medical Sciences, Shiraz, Iran; ^5^Department of Community Medicine, School of Medicine, Non Communicable Diseases Research Center, Fasa University of Medical Science, Fasa, Iran

## Abstract

**Background:**

There are shortcomings in nurses' adherence to ethical principles in practice. The present study aims to investigate the effectiveness of teaching nursing ethics via scenario-based learning and group discussion in nurses' adherence to codes of ethics and patients' satisfaction with nurses' performance.

**Methods:**

Using a quasiexperimental design, the present study employed questionnaires which measure nurses' compliance with nursing codes of ethics and patients' satisfaction with nursing care before, immediately after, and one month after intervention. The collected data were analyzed using the independent *t*-test, ANOVA, and chi-square test in SPSS v.22. The level of significance was set at *p* < 0.05. The nurses (*n* = 80) and patients (*n* = 160) from various units of two university hospitals in the south-west of Iran participated in the present study.

**Results:**

The pretest mean scores of the intervention and control groups in patient rights and patients' satisfaction with nursing care were not significantly different (*p*=0.07, *p*=0.21). Yet, there were statistically significant differences between the groups' mean scores as calculated immediately after (*p* < 0.001, *p* < 0.001) and one month after intervention (*p* < 0.001, *p* < 0.001).

**Conclusion:**

Employment of new approaches to teach nursing ethical principles improves compliance with nursing ethical codes and patients' satisfaction with nurses' performance.

## 1. Introduction

Advances in technology and expansion of nursing roles have led to complex ethical challenges in nursing [1]. Nurses have to work in a complicated and ever-changing system [2, 3]. Under these circumstances, hospitals must provide the suitable conditions for effective patient care combined with respect for patients' rights. Proper observance of patients' bill of rights leads to patient satisfaction, a better relationship between the staff and the patient, and a faster recovery [4]. Additionally, by adhering to professional ethical codes, nurses show their commitment to their professional duties: protecting the patient and providing care which will result in recovery [5].

Patients' satisfaction with their nursing care is an index of the quality and effectiveness of a healthcare system. Patient satisfaction is a mental and unique perception which is influenced by various factors, including nurses' manner of providing care [6] and respect for patients' rights [7, 8]. As respect for patients' rights is vital to providing compassionate and ethical care, nurses who are not familiar with ethical concepts cannot cope with professional challenges [4].

Adherence to ethical values, including respect for patients' rights and moral virtues, combined with clinical competence represent ethical nursing care [7]; however, there are reports of unkind and unjust care for vulnerable and senior citizens [8]. Additionally, research shows that nurses' degree of compliance with ethical principles is not satisfactory and nurses are not capable of applying their ethical knowledge in practice [3].

The best way to guarantee adherence to ethics in nursing practice is to introduce courses on professional ethics into the nursing curriculum [9]. Since nurses face ethical challenges in their daily practice, acquisition of ethical knowledge and adherence to the principles of professional ethics are an integral part of the nursing profession. An awareness of ethical principles makes for competent and ethical nurses who are capable of providing effective, satisfactory care [3, 10].

Education in ethical principles acts as a powerful antidote to issues stemming from ethical distress and consequently fortifies nursing [11]. However, the results of several studies in Iran show that nurses are not adequately familiar with ethical codes and do not comply with them in medical centers [3, 12–14]. In the study of Osingada et al., nurses' knowledge of ethical principles is found to be unsatisfactory and 93% of the interviewed nurses state that continuing nursing workshops is necessary [11]. In another study, only 25% of nurses are found be familiar with ethical codes [15]. Several studies also report that the current programs for education in ethics are not satisfactory [16, 17].

There are many ways of providing education in ethics, including lecturing, workshops, seminars, distance education, and group discussion [18]. The traditional methods of education, e.g., lecturing, can prove useful for transfer of large amounts of information over short periods; yet, in such approaches, learners play a passive part in their education and do not develop the skills required for problem-solving, decision-making, and critical thinking [19]. Therefore, there is need for active learning strategies to improve the outcome of education in ethics [20]. In their study, Chao et al. use a combination of interactive approaches—lecturing, videos, and group discussion—to teach professional ethics to nurses. The results show that an integrated approach can prove effective in increasing nurses' ethical awareness, decision-making, and analytical skills [21].

Another modern learning method is learning based on clinical scenarios [22]. Studies show that uncomplicated scenarios can contribute to nurse education and evaluation [23, 24]. The study of Uysal shows that scenario-based learning facilitates learning and retrieving knowledge [25]. Similarly, the study of Whyte et al. shows that simulated scenarios can be used for an effective evaluation of nurses' clinical performance [26]. Despite the potentially great contribution of teaching ethics via scenarios combined with group discussion, not many studies have addressed the effectiveness of educational interventions in enhancing nurses' adherence to patients' rights and patient satisfaction. Accordingly, the present study aims to evaluate the effectiveness of teaching nursing ethics via scenarios combined with group discussion in increasing nurses' adherence to ethical codes and patient satisfaction. The aim of this study was to measure the effectiveness of teaching nursing ethics via scenarios and group discussion in increasing nurses' adherence to ethical codes and patients' satisfaction with nurses' performance.

## 2. Methods

The present study is a quasiexperimental work conducted in the adult internal and surgical, CCU, post-CCU, emergency, delivery, pediatric, and hemodialysis wards of university hospitals located in the south-west of the province of Fars, Iran, between September and November 2018. The study population consisted of practicing nurses and hospitalized patients.

Based on the study of Hassanpoor et al., with a confidence level of 95% and power of 80% [27], the sample size was initially set at 27 subjects in each group which was raised to 40 subjects in view of the possibility of a 20% sample loss. To choose the subjects, the researchers acquired a list of the nursing staff and then used a random number table to select 80 nurses from the list. Subsequently, the nurses were randomly divided into a control and an intervention group: initially, the nurses were put in two homogeneous groups according to the variables of age, gender, and highest academic degree; next, a random number table was used (based on whether the last digit of the file number of each nurse was odd or even) to divide them into two 40-member groups.

Inclusion criteria for nurses were as follows: a bachelor's degree or above in nursing; at least one month experience of professional practice; not being in a nursing ethics workshop at the time of the study; and being willing to participate in the study.

Exclusion criteria for nurses were as follows: missing more than two sessions of the intervention and being uncooperative in the educational sessions.

Inclusion criteria for patients were as follows: a high school diploma or above; being familiar with one nurse; and at least 72-hour hospitalization in the ward.

Exclusion criteria for patients were as follows: unwillingness to participate.

Data were collected using two questionnaires which measured compliance with ethical codes and patient satisfaction. The former is a standardized scale approved by the ethics committee of the policy council at the Ministry of Health and Medical Education. The reliability and validity of the scale have been confirmed in various studies of nurses' and patients' perspectives [13]. The scale of compliance with ethical codes consists of 30 items which address the domains of respect for patient rights (13 items: 1–7, 13, 15, 17, 24, 25, and 26), professional commitment (14 items: 8–12, 14, 16, 18–23, and 27), and improvement in quality of care (3 items: 28–30). Scoring is based on a 5-point Likert scale: always = 5; usually = 4; occasionally = 3; rarely = 2; and never = 0. The score range is between 30 and 150, with higher scores indicating nurses' greater compliance with nursing ethical codes from patients' perspective. In the present study, the test-retest reliability of the scale was measured on 50 patients with a 2-week interval. The value of Cronbach's alpha was found to be 0.89.

The level of the patients' satisfaction with the nursing care was measured with a researcher-made questionnaire. The content validity of the questionnaire was evaluated by 15 nursing professors; the results showed that the questionnaire had satisfactory content validity: S-CVI (average) = 0.96 and I-CVI of each item was above 0.87. To test the convergent validity of the instrument, the researchers had 100 patients hospitalized in various wards complete the researcher-made questionnaire and the patient satisfaction instrument (PSI) simultaneously. PSI is a standardized scale initially developed by Risser in 1975 for evaluation of patients' satisfaction with their nursing care. The internal consistency and construct validity of the scale have been verified [28]. Also, the validity and reliability of the translated version of the instrument have been tested and confirmed in Iran [29, 30]. The results of the convergent validity test showed that the degree of relevance between the scores obtained with the two questionnaires to be high (r-0.78, *p* < 0.001). To measure the consistency of the patient satisfaction questionnaire, the researchers applied the test-retest method: the questionnaire was completed twice by the same 50 patients with a two-week interval. The total interclass correlation coefficient (ICC) of the questionnaire was found to be 0.95. The reliability of the instrument was measured via a calculation of its Cronbach's alpha: 100 patients completed the questionnaire and the results showed that it had an internal consistency of 0.90. The patient satisfaction questionnaire consists of 23 multiple-choice questions. For each question, there are 5 choices which are scored from 1 to 5: always = 5; often = 4; occasionally = 3; rarely = 2; and never = 1. The maximum score is 115.

Having acquired permission to conduct research from the ethics committee and department of research of the university, the researchers introduced themselves to the president, nurse manager, and head nurses of the hospital. First, the nurses' shift works were checked and the necessary arrangements were made with the supervisory office and head nurses to avoid interference with the educational intervention. Next, the nurses were introduced to scenario-based education, group discussion, and the benefits of learning nursing ethical principles. The educational sessions were held in the conference hall of the hospital. The questionnaires were given to the patients of each of the nurses in the control and intervention groups. The subjects were selected via the convenience sampling method. The educational intervention consisted of eight 4-hour sessions of education in ethical principles using clinical scenarios and group discussions. At each session, various ethical challenges were put forth and the participants discussed ways of dealing with them. The questionnaires were completed by the patients in the control and intervention groups immediately after and one month after intervention.

The collected data were analyzed using descriptive statistics and inferential statistical tests (*t*-test and chi-square) with a 95% confidence interval in SPSS v. 22. The normality of the data was examined via the Kolmogorov–Smirnov test. The Wilcoxon and Mann–Whitney tests were used for intragroup and intergroup comparisons, respectively. The significance level was set at *p* < 0.05. The research plan of the present study was approved by the ethics committee of the university. All the participants were informed about the objectives of the study. All the participants signed the informed consent form. The participants were assured of anonymity and confidentiality of their information.

## 3. Results

Of the 80 participating nurses, the majority (64%) were female and had a bachelor's degree (100%). The nurses' ages ranged from 22 to 65 years, with the mean being 29.36 ± 7.52. None of the nurses had participated in an ethics and professional values workshop. Of the 160 patients in the study, the majority (73.75%) were female. The mean and standard deviation of the patients' ages were 36.21 ± 9.32. 61.88% had an associate degree and 38.12% had a bachelor's degree. The results of the present study showed that, in the pretest stage, only 49.11% of the nurses were perceived to respect their patients' rights from the patients' perspective. Yet, after the intervention, the rate of respect for patients' rights increased to 77.9%.

The results of the *t*-test showed that there was not a statistically significant difference between the pretest mean scores of the control and intervention groups in compliance with ethical codes from the patients' perspective (*p*=0.07). However, according to the results of the chi-square test, the control and intervention groups had significantly different mean scores immediately after and one month after intervention. The compliance scores of the nurses in the intervention group were significantly higher than those of the control group as measured immediately after intervention from patients' perspective (*p* < 0.001, *p* < 0.001) (Table 1).

Of the 13 items related to the domain of respect for patient rights, “respect for patients' beliefs” was the most observed (2.62 ± 1.10) and “respect for patient's right to change nurse” was the least observed principle (2.20 ± 1.02).

Of the 14 items related to the domain of professional commitment, “providing an honest explanation in case of errors in nursing measures” was the most observed (2.77 ± 0.79) and “providing proper and timely care to alleviate patients' pain and suffering” was found to be the least observed principle (2.20 ± 0.90).

The 3 items related to the domain of improvement in quality of care (items 28, 29, and 30) were earning patients' trust by providing professional care, participating in care and treatment workshops, and encouraging families to support patients emotionally. The mean score obtained for each item was 2.36 ± 0.93, 2.31 ± 0.85, and 2.36 ± 0.93, respectively, which were rather equal.

With regard to patients' level of satisfaction with nursing care, the results of the *t*-test showed that the difference between the pretest satisfaction mean scores of the two groups was not statistically significant (*p*=0.21). However, based on the results of the chi-square test, the control and intervention groups had significantly different satisfaction mean scores immediately after and one month after intervention: the satisfaction mean score of the intervention group as calculated immediately after intervention was significantly higher than that of the control group (*p* < 0.001, *p* < 0.001) (Table 2).

## 4. Discussion

The results of the present study show a significant increase in nurses' respect for patients' rights as perceived by patients after intervention. Also, the intervention group's scores in compliance with nursing ethical codes and satisfaction with nursing care from patients' perspective were significantly higher than those of the control group immediately after intervention. Currently, there has not been much research into the effectiveness of scenario-based learning and group discussion combined in improving nurses' observance of patient rights and patient satisfaction. Accordingly, the literature mentioned below is only fairly close to the present study in subject.

According to one study, nurses' compliance with ethical codes is average which indicates the need for nurses' participation in organized and continuous professional ethics workshops [3]. In the study of Basiri Moghdam et al., nurses' extent of respect for patients' rights is greater than in the present study which can be attributed to the satisfactory awareness of the majority of the nurses with patients' bill of rights in the former study [31]. In their study, Vaskooei Eshkevari reported average levels of satisfaction with nurses' extent of respect for patients' rights [32]. The results of the study of Nikbakht et al. show increased compliance with ethical codes from the patients' perspective after intervention [33]. According to studies, all nurses and other members of a healthcare team are expected to consider the various aspects of ethical codes, beneficence, and maintaining an effective relationship. However, certain individual and organizational factors adversely affect the process of providing care and hamper the provision of ethical care by healthcare professionals. Thus, education alone cannot guarantee compliance with ethical principles in care and there is need for strategies to eliminate barriers to provision of ethical care and improvement of culture in health organizations [7, 8].

One of the reasons for nurses' failure to fully adhere to patients' bill of rights is their inadequate familiarity with the bill [33]. In the present study, the difference between the pretest and posttest levels of respect for patients' rights was statistically significant. In the study of Yoo and Park, case-based learning improves nurses' problem-solving skills more than lecturing [34]. According to the study of Endacott et al., simulation can contribute to nurses' clinical decision-making abilities [35]. In the present study, education in patient rights via scenarios and group discussions improved the hospital nurses' observance of patient rights: after the workshop, the mean total score in respect for patient rights was higher.

In the present study, the item “respect for beliefs” from the domain of respect for patient rights was found to be the most highly observed. This can be attributed to nurses' satisfactory knowledge and awareness in this area: studies show that nurses' awareness of respect for patients' beliefs is the highest [3, 36]. In their comparison of codes of ethics of nurse associations in England, Ireland, the Netherlands, and Poland, Dobrowolska et al. conclude that they all treat respect for patient rights as a top priority in nurses' professional performance [37]. Nurses are expected to provide respectful, competent, and unbiased care [38]. Yet, in another study, nurses are found to be inadequately familiar with patient rights and the researchers conclude that healthcare providers need workshops on patient dignity [1].

In the present study, the item “respect for patient's right to change nurse” from the domain of respect for patient rights was found to be the least observed, while in 36% of cases, patients' freedom of choice and autonomy are respected and only 17% of the patients are given the right to change their doctor or nurse [32].

In the present study, the intervention group's level of respect for patient privacy increased after intervention. In a study of professional values in healthcare, the items of patient privacy and confidentiality are found to be of high priority to nurses [1]. Nasiriani et al. report that despite the importance of respect for patient privacy, the majority of inpatients are dissatisfied with nurses' performance in this area [39]. Another study reports the status of respect for patient privacy to be satisfactory [32] which is consistent with the findings of many other studies. These inconsistencies can be due to differences in locations and cultures of study subjects. Since most studies report high scores regarding respect for patient privacy, it can be concluded that this dimension of patient rights lies at the core of patients' bill of rights and nursing professional ethics.

In the present study, from the patients' perspective, access to an efficient system for dealing with complaints improved after intervention. Observance of patients' right to have access to such a system has been increasing [40], which seems to be the result of an increase in the awareness of healthcare teams. However, as providers of healthcare, nurses need to be made better aware of patient rights [41].

In the present study, from the domain of professional commitment, the code of “providing an honest explanation in case of errors in nursing measures” was found to be the most highly observed. Research indicates that nurses and other members of hospital staff are liable to make mistakes as no one is infallible; however, many studies show that over 40% of such errors are never reported mostly because of a fear of being penalized. Nurses' culture and attitude toward the policies of the organization where they are practicing influence the rate of reported errors [42]. It can be concluded that in case of an error in their practice, nurses are faced with many barriers to tell the truth. This can explain the average level of compliance with “providing an honest explanation in case of errors in nursing measures” in the present study.

In their study, Jouzi-arkawazi et al. report that only a small number of nurses are sufficiently familiar with patients' bill of rights [43]. The findings of the present study show that compliance with ethical codes in the domains of “providing timely care” and “providing correct and skillful care in times of pain and suffering” was less than average. According to one study, providing timely and proper care is one of the most important dimensions of observance of patient rights [44]. According to the study of Khodaveisi and Hosni, the degree of compliance with such ethical codes as respect for patients, reporting errors, confidentiality, and responsibility is average or occasionally satisfactory, which is consistent with the findings of the present study [45].

In the present study, the level of satisfaction of the patients in the intervention group was significantly higher immediately after intervention. In the pretest stage, the patients' satisfaction with the quality of nursing care was average, which is consistent with the findings of the studies of Joolaee and Freitas [29, 46]. The increase in the patients' satisfaction following the intervention proves the effectiveness of education in ethics via scenario-based learning and group discussion. The results show a slight decrease in the scores of the intervention group as measured one month after intervention. This can be attributed to the impact of time on learning and suggests the need for continuing education.

In the present study, it was possible that the subjects in the control and intervention groups were practicing in the same hospital ward, and, therefore, there was a chance of transfer of the educational content from the intervention group to the control group. To minimize the impact of that limitation on the results of the study, the researchers did not inform the subjects about which group they had been placed in. However, the researchers were not able to fully eliminate that possibility.

## 5. Conclusion

The results of the present study show that, in the domain of nursing ethical codes, educational interventions using modern approaches can improve nurses' observance of patient rights, thereby increasing patient satisfaction. It is recommended that educational programs which use scenario-based learning and group discussion in the fields of nursing ethics and patient rights be designed not only for nurses but also nursing students, so that the latter can acquire the necessary awareness and competences with regard to professional ethics before entering the world of professional clinical practice.

## Figures and Tables

**Table 1:** A comparison between the mean scores of the control and intervention groups in compliance with nursing ethical codes as measured in three stages: before, immediately after, and one month after intervention.

Group	Stage			
	Before	Immediately after	One month after	*p* value^*∗*^

Intervention	73.67 ± 14.46	116.90 ± 13.84	113.69 ± 13.18	<0.001
Control	79.06 ± 22.63	78.08 ± 6.91	76.81 ± 6.56	0.394
*p* value^*∗∗*^	0.075	<0.001	<0.001	

^*∗*^Repeated measure; ^*∗∗*^*t*-test.

**Table 2:** A comparison between the satisfaction mean scores of the control and intervention groups as measured in three stages: before, immediately after, and one month after intervention.

Group	Stage			
	Before	Immediately after	One month after	*p* value^*∗*^
Intervention	56.27 ± 9.86	86.22 ± 10.9	83.70 ± 11.31	<0.001
Control	58.58 ± 15.85	57.84 ± 6.12	57.59 ± 6.40	0.510
*p*-value^*∗∗*^	0.219	<0.001	<0.001	

^*∗*^Repeated measure; ^*∗∗*^*t*-test.

## Data Availability

The data used to support the findings of this study are available from the corresponding author upon request.
